# We Hold Ourselves Accountable: A Relational View of Team Accountability

**DOI:** 10.1007/s10551-021-04969-z

**Published:** 2021-11-18

**Authors:** Virginia R. Stewart, Deirdre G. Snyder, Chia-Yu Kou

**Affiliations:** 1 University College Dublin, Dublin, Ireland; 2 Providence College, Providence, RI USA; 3 Cranfield School of Management, Cranfield University, Bedford, UK

**Keywords:** Collective, Commitment, Felt accountability, Group, Longitudinal, Measure, Performance, Relational accountability, Team, Trust

## Abstract

Accountability is of universal interest to the business ethics community, but the emphasis to date has been primarily at the level of the industry, organization, or key individuals. This paper unites concepts from relational and felt accountability and team dynamics to provide an initial explanatory framework that emphasizes the importance of social interactions to team accountability. We develop a measure of team accountability using participants in the USA and Europe and then use it to study a cohort of 65 teams of Irish business students over three months as they complete a complex simulation. Our hypotheses test the origins of team accountability and its effects on subsequent team performance and attitudinal states. Results indicate that initial team accountability is strongly related to team trust, commitment, efficacy, and identifying with the team emotionally. In established teams, accountability increases effort and willingness to continue to collaborate but did not significantly improve task performance in this investigation.

## Introduction

Accountability is prevalent in the ethics literature because it makes people attend to the relevant prescriptions for conduct and provides a mechanism for the social control of behavior in accord with those prescriptions (Quinn & Schlenker, 2002). Practitioners and academics alike treat accountability as a means of directing and correcting individual and organizational efforts and performance and encouraging socially responsible behaviors. Accountability may be thought of as the “adhesive that binds social systems together” (Frink & Klimoski, 1998, p. 3) and as such is not only a fundamental principle of the organizational sciences, but is also a necessity for the effective operation of enterprise (Frink & Klimoski, 1998; Lerner & Tetlock, 1999).

Traditional, formal accountability systems are fading in many quarters as authority-based relationships decline (Moncrieffe, 2011). The ongoing transformation in the basic organization of work has resulted in a less hierarchical structure and the emergence of teams as the core building blocks of organizations (O’Neill & Salas, 2018), which calls for an urgent reexamination of the inherent nature of accountability. In particular, this change necessitates a better understanding shared accountability (Bergsteiner, 2012; de Leede et al., 1999), or how ‘we’ hold ourselves accountable.

Our paper joins the shift away from studying accountability as an objective external feature of the context, i.e., reporting and surveillance systems or obligations to stakeholders, and instead develops a view of accountability that is derived from relationships and social exchanges and is collectively lived and breathed (Frink & Klimoski, 2004; Gefland et al., 2004; Painter-Morland, 2006). The human core of accountability is particularly relevant in times of turmoil such as the global health pandemic. An orderly environment can be effectively dealt with by formal measures, but professionals across the globe report that flourishing in chaos requires the renewed focus on human purpose, fairness, ethics, collaboration, and relationships. In this context, effective teaming has become a ‘life raft’ keeping organizations and their talent afloat during COVID-19 (Deloitte, 2020, p. 24).

We define team accountability as team members’ shared expectations of being held answerable for their common actions or decisions (Frink et al., 2008; Kou & Stewart, 2018; Lerner & Tetlock, 1999). These expectations arise through an interdependent process of group members interpreting formal accountability mechanisms, introducing felt obligations, shaping routines and communication patterns, and nurturing the trust, loyalty, and commitment that encourage or curtail member behaviors. Our proposed conceptualization of team accountability includes an important moral dimension because team members are not only accountable to external authorities, but are also accountable to and for each other, making them moral actors with an obligation to serve the interests of the team (Clark & Brown, 2015; Robinson, 2015).

Such an approach applies a relational understanding of accountability to the team context. This perspective spotlights the relational context within which responsibilities and duties develop and focuses attention on the dynamic network of interactive relationships within which individuals and organizations are embedded in the business environment (Painter-Morland & Deslandes, 2017). An order emerges over time through the interactions of individuals who participate in the team. This developing sense of reciprocal responsibility, commitment, and shared purpose may resist being codified as formal rules and procedures, but it shapes the normative backdrop against which the actions and decisions of the team play out.

The relational approach to team accountability provides support for treating the concept as an emergent team phenomenon. Emergent constructs form over time through a synthesis of the interactions of team members (Cronin et al., 2011; Marks et al., 2001). In the context of teams, emergent phenomena ‘bubble up’ from lower-level constituent properties that exist in the form of individual and dyadic behaviors (Waller et al., 2016, p. 565). This means that team accountability cannot be examined before the team has been formed or has worked together. Team accountability in our view exemplifies ‘strong’ emergence as it arrives from lower-level parts but it exceeds their sum, continues over time, is substantial enough to be felt and experienced by team members, and can exert downward causal forces that affect individual team members and their interactions.

This paper advances the concept of team accountability through four approaches. First, we review and integrate ideas from relational accountability and team dynamics to provide a cross-disciplinary conceptualization of team accountability. In this conceptualization, the primary accountability actor is the collective rather than the individual, and the audience to whom the team is accountable includes its members.

Our second contribution is to develop a working measure (Team Accountability Questionnaire, TAQ hereafter) that addresses limitations in existing accountability measurements (Hall et al., 2017; Kou & Stewart, 2018). This 8-item measure was developed using a U.S. business student sample (Study 1a) and validated using senior leaders in the U.K. (Study 1b) and an online paid subject pool from the U.S. (Study 1c).

We then used the measurement tool to follow 65 teams of Irish business students for three months as they completed a complex business development simulation (Study 2). Based on our results, our third contribution is to establish collective team accountability as a coherent emergent state and to demonstrate its relationship with team trust, commitment, affective identification, and team efficacy. Our fourth contribution addresses practical concerns about collective accountability. Presumably getting collective accountability ‘right’ matters because it should inhibit social loafing, increase performance, and reduce turnover intentions, all outcomes that are valued by organizations. We therefore examine whether team accountability indeed predicts collective effort, task performance, and willingness to work together again (Study 2).

## Theoretical Background

An often made point in the ethics literature and observations of business practices is that even the most carefully conceived accountability mechanisms do not always lead to accountable behaviors by individuals or organizations (Mansouri & Rowney, 2014). For instance, individuals occupying comparable work environments, with equivalent demands and expectations, can perceive increased accountability as coercive and oppressive, while others in the same context welcome accountability and engage proactively with performance targets (Ogden et al., 2006).

Acknowledging this reality, many researchers have advocated a phenomenological view of accountability, which argues that accountability is best viewed as a subjective interpretation, or a state of mind (i.e., felt accountability), rather than an objective state of affairs (Breaux et al., 2009; Frink et al., 2008; Gefland et al., 2004; Hall et al., 2017; Hochwarter et al., 2005; Hochwarter et al., 2007; Lanivich et al., 2010; Wallace et al., 2011). Indeed, subjective interpretations are typically more important than the actual external context because these perceptions drive attitudes and behavior (Lewin, 1947). A supportive context is necessary but not sufficient for the development of team accountability (de Leede et al., 1999). For this reason, we examine team accountability through collective perceptions of accountability rather than through the existence of formal accountability mechanisms.

If team accountability is indeed a subjective interpretation of objective accountability mechanisms, what prompts these interpretations in a team context? We suggest that social interactions between group members give rise to a collective understanding of what accountability entails for the group. To support the criticality of relationships with team members in substantiating team accountability, we draw on recent work on relational accountability.

### Relational View of Team Accountability

Accountability is increasingly being seen through a wider lens that includes the social relationships and power dynamics that influence what accountability means in practice (Moncrieffe, 2011). A relational understanding of accountability proceeds from the assumption that the most meaningful normative duties and responsibilities resist legalistic formulation and codification and originates with the premise that we are primarily social creatures and that social relationships shape accountability (Painter-Morland, 2006, 2007, 2011). Being in a relationship and wanting to maintain it—i.e., relational responsiveness—is fundamental to the mindset of expecting to give an account. Relational approaches to accountability inquire how people experience accountability and how the quality of social relationships shapes accountability outcomes.

This understanding of accountability highlights the dynamic network of interactive relationships within which individuals and organizations are embedded (Beu & Buckley, 2001; Gefland et al., 2004; Johansen, 2008; McGrath, 1991), for it is “only within the context of particular relationships that any kind of account becomes meaningful and significant” (Painter-Morland, 2006, p. 93). This does not imply permissive subjectivism or relativism. Rather, the dynamic expectations that define moral duties and obligations within a relational context provide guidance. These expectations may be particularly salient at key milestones, such as the midpoint of a project (Gersick, 1991). Furthermore, the kinds of moral constraints that emerge in the context of specific relationships may arguably be more demanding than conventional accountability mechanisms. For example, the degree of peer agreement that an action is wrong decreases unethical behaviors far more than the presence of an organizational code of ethics (Kish-Gephart et al., 2010). Moreover, formal accountability mechanisms can paradoxically interfere with moral responsivity (Beu & Buckley, 2001), such as when accountability for outcomes leads to greater self-serving behaviors (Pitesa & Thau, 2013) or misallocation of limited funds (Adelberg & Batson, 1978).

According to the relational understanding of accountability, values result from key individuals within a particular system interacting with each other. These values represent an ‘emergent order’ which is the result of relational dynamics rather than the unilateral imposition of formal accountability mechanisms or moral ideals (Painter-Morland, 2006, p. 94). Everyday engagements between stakeholders enable actors to identify and refine the nature and extent of their moral obligations and shape mutual expectations (Painter-Morland, 2006, 2011). The need to belong is a core human motive (Baumeister & Leary, 1995; Deci & Ryan, 2000) and individuals are motivated to adjust behavior in order to gain or maintain membership in a group that has a high relational value (Leary, 2001). Specifically, among collaborators, these interactions create a tacit sense of reciprocal responsibility, loyalty, and common cause that shapes behaviors in a way that may not be formally articulated (Painter-Moreland & Deslandes, 2017). Thus, relational accountability not only provides a basis for a shared sense of propriety and morality, but also draws emotional as well as cognitive aspects of value (Robinson, 2015) and encourages behavior that fortifies continued group membership.

Applying these concepts to team accountability suggests that ongoing meaningful interactions between team members give rise to team accountability practices beyond any formal accountability mechanisms or individual attributes (Moncrieffe, 2011). From an ethics perspective, team accountability can be viewed as group members interpreting formal policies, introducing felt obligations, shaping interaction patterns, and providing the context within which team behaviors are supported or curbed. Such internal team interactions may be more demanding than external sources of accountability in terms of providing meaningful guidance, in part because members themselves contribute to the emergence of mutual accountability and to the rewarding or sanctioning of team members.

A relational understanding of accountability indicates that team accountability is an emergent team phenomenon. Emergent constructs are variable and dynamic and form over time through a synthesis of the interactions of team members (Cronin et al., 2011; Marks et al., 2001). Emergent team phenomena emanate from a series of behaviors of group members and include states such as collective cognition or emotions as well as behavioral patterns and power structures. Each manifestation of team accountability will be unique because the lower-level components interact in a dynamic fashion. To use the language of emergence, team accountability is ‘radically novel’ (Waller et al., 2016) because it can neither be predicted from individual team member accountability nor reduced to component parts, and exists at a level higher than individual team members (Kozlowski & Klein, 2000). This implies that studying team accountability requires an explicit focus on group dynamics.

A hallmark of an emergent state is its coherence over time (Marks et al., 2001; Waller et al., 2016). If team accountability ebbs and flows in response to internal and external dynamics, we would still expect it to endure as a global team property. Coherence and endurance do not necessarily imply that group members’ perceptions of accountability converge, but rather that team members are able to collectively recognize and experience team accountability in a persistent way for a nontrivial time period as they perform interdependent organizational tasks. With awareness of team accountability comes potential feelings of agency by group members concerning their ability or desire to act to change accountability patterns. This means team accountability is not only the bottom-up product of team member interactions over time, but in theory exerts top-down influence on ongoing group member interactions.

### Hypotheses Development

At the start we defined team accountability as team members’ expectations of being held answerable for their common actions or decisions. Based on a relational understanding of accountability we made the argument that expectations of being answerable as a team are in large part the product of ongoing group member interactions in which formal accountability mechanisms are interpreted and internalized by the team. While individuals contribute to these dynamics, team accountability is a global phenomenon, unique and irreducible to individual members.

#### Co-emergence of Team Accountability

During the formative stage of team development the continuous social dynamics between group members will naturally shape team states in addition to initial accountability expectations. We suggest that in the context of studying primordial team states, it is appropriate to examine co-variation rather than causation (Costa et al., 2014; Gucciardi et al., 2018). Therefore, we first identify the constructs with which team accountability ought to co-emerge during its infancy and defer making precise causal statements about the consequences of team accountability until enough time has passed for a team to accumulate a series of meaningful performance episodes.

Within the realm of related team states that encircle nascent team accountability, the most primary must certainly be the *trust* felt within the team. In abstract, accountability and trust both refer to behavioral expectations held by parties in a relationship and evolve based on interpersonal interactions and group dynamics (Costa et al., 2018). Trust and accountability can both be seen as a moral phenomenon that occurs in the context of team relationships (Cohen & Dienhart, 2013). Trust, however, is distinct from accountability because it refers to an actor’s willingness to be vulnerable to the actions of another party, irrespective of the ability to control that other party (Mayer et al., 1995).

We anticipate that when a team starts to collaborate, accountability and trust build on and reinforce another. Trust may be ready to be activated on newly formed teams due to preexisting conditions such as strong identification with the group, imported expectations about roles or skills, or anticipating future professional interactions (i.e., ‘swift trust’, Kroeger et al., 2021; Meyerson et al., 1996). Trust may also develop incrementally through interpersonal interactions (Costa et al., 2018). In both forms, we expect that trust should help convert individual actions into coordination and collaborative team effort (Dirks, 1999; Meyerson et al., 1996), thereby positively influencing perceptions of accountability shared among team members.

An alternative perspective is that when initial trust is high, there is less need to invest in monitoring the relationship (Ammeter et al., 2004). However, we suggest that when trust is high, teammates will still feel morally accountable to each other and behave cooperatively, independently of whether or not they are held formally accountable (De Cremer et al., 2001). This suggests that trust and team accountability are not operating along a single continuum (Ehren et al., 2019).

Actually being accountable as a team can also ensure that trust becomes an established feature of the team (Ehren et al., 2019). Initial efforts to establish accountability will signal reliability and acceptance of vulnerability to interdependencies as well as emphasize collective well-being. Indications of teammates' competence, benevolence, and integrity should positively influence evaluations of collective trustworthiness (Mayer et al., 1995) which will encourage trusting behaviors such as relying on others' skills or judgments or delegating tasks to as yet unproven colleagues.

##### H1a

Team accountability is positively related to initial team trust.

In addition to showing a positive relationship with trust, we anticipate that team accountability will emerge in tandem with a sense of oneness as a team (Ashforth & Mael, 1989; Zhang et al., 2014). This affective *identification* with a team is associated with feelings of pride and joy (Johnson et al., 2012), greater belongingness (Ashforth & Mael, 1989; Dutton et al., 1994), and deeper internalization of and contribution towards the goals of the workgroup (Mael & Ashforth, 1992). Even among team members with conflicting goals a sense of team identification allows members to share their perspectives and work together to maximize team performance (De Cremer & Van Vugt, 1999). Thus, higher affective identification for the team is likely to instill a stronger sense of ‘we’ that will boost internalization of accountability expectations as team members work together to overcome conflicting goals and engage in collective actions to drive performance.

A spirit of unity and togetherness should support the emergence of relational accountability within the team. When team members connect on a positive emotional level and where being a member of the group is valued (Riketta & Van Dick, 2005), the salience of maintaining healthy relationships with other team members will be higher. However, fault lines—potential team division into subgroups based on the alignment on one or more social categories—can make it very difficult for a superordinate team to emerge in the presence of stereotyping and in-group favoritism (Bezrukova et al., 2009; Jehn & Bezrukova, 2010; Thatcher, 2011). In teams with active subgroups and weak team-level attachments, relational accountability should more naturally gravitate around subgroups. Drawing attention to shared accountabilities could help bridge differences in such situations by reinforcing the interdependence of subgroups. Therefore, we offer the following hypothesis.

##### H1b

Team accountability is positively related to initial team identification.

The third dynamic relevant to understanding team accountability during the early stage of team development is *commitment*. Acceptance and belief in the team’s goals and a willingness to exert effort on behalf of the team should support the growth of team accountability expectations. The more determined a group is to do its best, the more likely the group is to monitor its performance against their agreed upon objectives and expect to explain lapses in performance relative to team goals. Commitment has further been shown to increase information sharing within teams (Aubé et al., 2014; Liu & Li, 2018), which should encourage communication norms of keeping each other informed about task progress. Beyond facilitating information flow and performance tracking, commitment to a higher purpose also stimulates the development of positive working relationships and citizenship behaviors to achieve those objectives (Aubé & Rousseau, 2005; Bishop et al., 2000; Pearce & Herbik, 2004). These reasons lead us to anticipate an initial positive effect of commitment on accountability.

The countervailing argument is that team accountability expectations are a precursor to commitment. The general challenge of accountability is how to get the best out of people—how to secure their commitment to desired behaviors (Bergsteiner, 2012). Expecting to be held answerable to a relevant audience is supposed to increase a team’s persistence and effort in the desired direction. Under this premise, shared accountability expectations will motivate teams to allocate their efforts and resources in ways that allow them to address expectations (Katzenbach & Smith, 1993). For example, when a group is held collectively accountable for making quality decisions, members focus their energy on exchanging more information and achieving shared outcomes (Liu & McLeod, 2014; Scholten et al., 2007). Given the support for mutual causality, we predict a positive relationship between accountability and commitment at the launch of a team.

##### H1c

Team accountability is positively related to initial team commitment.

*Team efficacy* is the fourth state we suggest will emerge along with team accountability. When a team believes that it has sufficient ability to complete its assigned tasks and to do them well it is more likely to anticipate accounting for achievements rather than failures (Gully et al., 2002; Zacarro et al., 1995), which would lead to embracing shared responsibilities. Evidence from the military demonstrates that a team’s belief in its ability to solve problems and make good decisions improves social cohesion (Hirshfield and Benerth, 2008) which should facilitate the relational connections needed for team accountability to emerge.

In the early stages of team development, accountability should increase a team’s belief in its ability to do the work it has taken on. Team accountability contributes to perceptions of team efficacy through its effects on coordination and cooperation. Expecting to account as a group and to each other involves taking responsibility for decisions, monitoring progress, explaining choices, and being able to count on members to meet their commitments. These patterns of behavior are likely to strengthen the team’s beliefs in its capabilities (Watson et al., 2001).

##### H1d

Team accountability is positively related to initial team efficacy.

We suggest that by the halfway point between the beginning and final team deadline, team accountability should be tangible enough to predict subsequent outcomes of interest to organizations. Team members should have established a communal history, social and task interaction patterns are better established, and there should be fewer unknowns and more givens (McGrath, 1991). The basic configuration into which the team's members are organized and maintenance activities are executed should be assembled (Gersick, 1988, 1991) and having to account for team efforts will be more salient at the midpoint than at the start, as will the potential for rewards and penalties. Awareness of time becomes acute at the midpoint and fear of running out of time can create a ‘jolt of urgency’ (Gersick, 1991, p. 27). This heightens performance pressures and raises concerns about group image. Characteristic responses include checking external requirements, seeking outside assistance, making temporal commitments, and regulating the flow of task and interpersonal interactions (Chang et al., 2006; Gersick, 1988), each of which highlights the salience of collective accountability. We therefore anticipate that by the midpoint of most collaborative projects, a team will have interacted sufficiently for a sense of collective accountability to mature from a generic meaning into one that is uniquely situated within that particular team and organizational context (McGrath, 1991).

#### Consequences of Team Accountability

Understanding the relationship between team accountability and team performance is critical because organizational teams are formed to deliver on stakeholder objectives (O’Neill & Salas, 2018). Productivity includes the behaviors employed in reaching targets (Klimoski & Mohammed, 1994) as well as the growth potential of the team (Bell & Marentette, 2011; Hackman, 1987). Without viability, members will eventually burn out even if a team is productive. The following section therefore proposes relationships between team accountability and task performance and effort, as well as the long-term sustainability of the team.

The positive effects of accountability mechanisms on team performance have been explored primarily in experimental contexts with nominal teams subjected to various accountability conditions. Accountability has often been investigated as a potential antidote to process loss in group decision-making. For example, when teams are told they will be held accountable for how they make decisions, they exchange more information (Liu & McLeod, 2014) and more often choose the correct alternative (Scholten et al., 2007). Group members expecting to be collectively responsible display less groupthink than control groups. In particular, accountability staves off excessive consensus-seeking, results in a better power balance within the group, and produces less risky decisions (Kroon et al., 1991). Outside the laboratory environment, Wallace and colleagues report better sales and customer service in restaurants with higher shared accountability in management staff, so long as they also felt empowered (Wallace et al., 2011). The relational nature of team accountability should focus the team on achieving collective goals for reasons in addition to—or in spite of—formal accountability systems. Thus, we predict a positive relationship between accountability and team task performance.

##### H2

Midpoint team accountability predicts higher endpoint performance.

Performance measures contain variance attributable to factors other than teamwork such as availability of resources or industry shocks and are usually not diagnostic because they may not indicate potential underlying causes (Cannon-Bowers & Salas, 1997). Process measures address the strategies and behaviors deployed to reaching desired outcomes. Team process-based performance may include, among others, levels of collective effort expended or the quality of interpersonal relationships (Klimoski & Mohammed, 1994). We anticipate that feelings of being morally obligated to another should create a shared motivational reality which influences the direction, intensity, and persistence of team behavior over time (Park et al., 2013). Team accountability should therefore both increase goal-directed effort and reduce social loafing, free-riding and other tendencies to reduce efforts or escape responsibilities (Karau & Williams, 1993; Latané et al., 1979). We therefore suggest:

##### H3

Midpoint accountability predicts greater endpoint effort.

The sustainability and growth potential of a team—i.e., team viability—is an essential measure of effectiveness for groups that have longer lifecycles (Bell & Marentette, 2011; Hackman, 1987). Teams with a stronger sense of shared accountability should be better positioned to stay together in the long run than those with weak feelings of mutual obligation because they have developed work processes and communication norms that facilitate successful performance. Healthy team accountability behaviors should discourage burnout and divisive behaviors because members feel an obligation to help others and provide social and instrumental support. The nature of feeling accountable to each other further solidifies the sense of belonging to the group, which facilitates in-group bias and the desire to maintain membership in the group (Ashforth & Mael, 1989). Indeed, antecedents of team viability include denser emotional connections within the team and greater social integration (Balkundi & Harrison, 2006; Foo et al., 2006). This leads us to expect the following relationship:

##### H4

Midpoint team accountability predicts stronger viability.

In addition to impacting performance outcomes, we expect that accountability will positively influence relationship dynamics. We assume that when a team starts collaborating, accountability emerges hand in hand with other team dynamics which muddies causal effects. To identify the unique impact of accountability requires space for an accountability mindset to develop. Once an accountability mindset develops, it should be predictive of greater team trust and deeper commitment to the shared cause. These two attitudes are foundational to the functioning of the team and relevant throughout the life of the team:

##### H5a

Midpoint team accountability predicts greater endpoint trust.

##### H5b

Midpoint team accountability predicts greater endpoint commitment.

## Methodology

In order to test these hypotheses we first needed to address measurement issues. In their review of felt accountability in organizations, Hall and colleagues identified the lack of a validated instrument that captures answerability to audiences other than top management and the reliance on experimental conditions that may not capture the relational, dynamic, and perceptual nature of accountability as two main issues limiting accountability research (2017).

### Study 1a: Scale Development

Our research process therefore started by developing and validating a team accountability questionnaire that could work across organizational levels. In a conceptually valid measure of shared team accountability the primary accountability actor is the group (not the individual), the audience to whom the team is accountable includes its members, and the content assesses collective expectations about explaining and justifying team decision processes and performance, as well as team dynamics related to relational accountabilities. We followed deductive scale development processes to generate an initial set of items (Hinkin, 1998) because our definition of team accountability builds on the classic definition of accountability which focuses on the expectation of answering for behaviors and outcomes to a relevant audience (Frink & Klimoski, 1998; Lerner & Tetlock, 1999).

#### Item Generation

A search of the accountability literature identified 120 individual accountability items that could be shifted to the level of the team by changing the referent from ‘I’ to ‘we’ and by changing the content to reflect shared expectations (Chan, 1998). We whittled down this list to 22 items by prioritizing three scales developed with input from managerial samples (Doney & Armstrong, 1996; Mero et al., 2014; Ogden et al., 2006), a scale that has been used widely in the felt accountability domain (Hochwarter et al., 2005), and several checks of experimental manipulations of accountability (e.g., Pitesa & Thau, 2013). Through discussions with an expert on teams on content validity, we realized that our initial pool of items drawn from the *individual* accountability literature might not fully capture the ongoing *team* member interactions we had theorized were essential to formal accountability demands being interpreted and internalized. For that reason we wrote five additional items specific to team processes that would reinforce perceptions of being accountable to each other.

#### Measures

We also collected measures of team trust, identification, commitment, and viability so that we could examine the reliability of these measures prior to relying on them to test our hypotheses. Affective *identification* (Johnson et al., 2012) was measured with three items. Intragroup *trust* (Simons & Peterson, 2000) was measured with four items. Team *commitment* (Kirkman & Rosen, 1999) and *viability* (Marrone et al., 2007) were measured with five items each.

#### Sample

Eighteen teams of undergraduate business students enrolled in a required management course at a U.S. university pilot tested our accountability items. The students had been working together in randomly assigned teams of four or five members over the term to develop a video. The total sample size was 70 individual participants (45 percent women). Participants responded to an electronic survey link. Items were assessed by participants indicating the extent to which they agreed or disagreed with a given statement where 1 = strongly disagree to 7 = strongly agree.

#### Analyses

SPSS was used to evaluate the most suitable items from our list of 27 possible candidates. Redundant items and those that exhibited low inter-item correlations were discarded. We screened out accountability items that correlated too highly or not enough with team trust, identification, commitment, and viability. Corrected item-scale correlations were inspected and several more cuts were made to ensure retained team accountability items were representative of the whole theoretical domain. The eight selected items of the Team Accountability Questionnaire (TAQ) indicate a coefficient alpha (*α*) of 0.90. Item means and correlations with theoretically related scales are reported in Table 1. The provenance of each piloted item is provided in the Appendix.

**Table 1:** Descriptive statistics (Study 1a)

	*Mean*	*SD*	*N*	1	*(1)*	*(2)*	*(3)*	*(4)*	*(5)*	*(6)*	*(7)*	*(8)*	2	3	4	5
1. Team Accountability Questionnaire (TAQ)	5.85	.95	70	***.90***												
(1) We are accountable to each other as a team for our performance	6.10	1.19	70	.79	–											
(2) We are required to justify or explain our performance relative to team goals	5.17	1.45	70	.74	.54	–										
(3) We hold each other accountable for doing our individual work	6.07	1.22	70	.85	.64	.54	–									
(4) We take responsibility as a group for all of our decisions	6.03	1.17	70	.73	.51	.36	.52	–								
(5) In meetings we often explain to each other why we do certain things	5.70	1.12	70	.72	.59	.45	.61	.46	–							
(6) We monitor our team performance against our objectives	5.71	1.36	70	.75	.46	.58	.59	.42	.44	–						
(7) We keep each other informed of our progress	6.09	1.05	70	.78	.61	.42	.62	.75	.55	.48	–					
(8) We can count on members to deliver what they promise	5.94	1.33	70	.79	.57	.51	.71	.54	.42	.57	.49	–				
2. Team Trust	6.04	1.12	50	.72	.58	.53	.64	.63	.49	.44	.53	.55	***.94***			
3. Team Identification	5.74	1.35	50	.74	.59	.55	.74	.51	.57	.45	.59	.53	.66	.***93***		
4. Team Commitment	6.16	.84	55	.74	.67	.54	.58	.58	.46	.60	.59	.57	.70	.73	***.89***	
5. Team Viability	5.74	1.02	55	.69	.50	.52	.65	.53	.55	.52	.62	.41	.72	.84	.65	.***91***

All participants responded to the accountability items, but were randomly assigned a subset of the remaining measures to reduce fatigue; all correlations are significant at the 0.01 level (2-tailed); scale reliabilities are reported in bold font on the diagonal

We then used exploratory factor analyses (EFA) to examine the convergent and discriminant validity of the TAQ. Large samples are always beneficial; however, when factors are well defined or their number is limited, small sample size EFA can yield reliable solutions (de Winter et al., 2009). We specified a two-factor solution with oblique rotation and examined accountability and trust, accountability and commitment, accountability and identification, and accountability and viability.

#### Results

The accountability items loaded on their intended factor. Factor loadings for the accountability items ranged from 0.33 to 0.94 with an average of 0.65 across the EFAs. In all analyses there are 7 or more strongly loading team accountability items (0.50 or higher) which indicates a solid factor (Costello & Osborne, 2005).

### Study 1b: Scale Validation

To examine the internal structure of the TAQ and ensure it is appropriate for use in more traditional work settings and with mature professionals, the lead author distributed an electronic survey to all the senior leaders of a large multinational pharmaceutical company headquartered in the United Kingdom. This site was selected because its senior leadership had chosen accountability as one of their core leadership themes and were tracking efforts to integrate shared accountability for decisions and results into their daily activities. One hundred leaders provided complete responses for a completion rate of 78 percent, of which 71 percent had worked at the organization for more than a decade. Two-thirds were based in Europe and the remainder in North America.

#### Measure

Each respondent reported on the accountability behaviors of their particular leadership team. The eight items from the piloted questionnaire were slightly modified to suit the organizational context (i.e., ‘I am part of a leadership team that … is accountable as a team for our performance’). Response options ranged from 1 = strongly disagree to 5 = strongly agree. Coefficient alpha (*α*) of the measure is 0.86 in this sample.

#### Results

We conducted EFA with principal axis factoring. Results of the scree plot and eigenvalue-greater-than-one rule indicate that the measure is unidimensional, which matches the theoretical factor structure. The item with the highest loading is ‘I am part of a leadership team that is accountable for our performance’ which is appropriate for the rationale for the TAQ. All item loadings exceed 0.60 per Table 2. These exploratory results provide support for the internal validity of the TAQ in an applied setting with senior professionals.

**Table 2:** Exploratory factor analysis of TAQ (Study 1b)

*I am part of a leadership team that ...*		Factor Loading
1	Is accountable as a team for our performance	.80
2	Justifies or explains our performance relative to team goals	.73
3	Holds team members accountable for doing their individual work	.70
4	Takes responsibility as a group for our decisions	.65
5	Often explains to team members why we do certain things	.73
6	Monitors our team performance against our objectives	.65
7	Keeps members informed of our progress	.74
8	Can count on team members to deliver what they promise	.66
Eigenvalue		4.50
Variance		56.23

### Study 1c: Scale Validation

The purpose of Study 1c was to use confirmatory factor analyses (CFA) to examine the convergent and discriminant validity of the TAQ. 217 participants were recruited from an online commercial survey pool (Mechanical Turk) and received $1.00 in compensation for completing our questionnaire. The average age in the sample is 39 years. Slightly more than half of the participants were female and 28 percent consider themselves minority. Participants have been with their current employer for an average of eight years and with their primary team for an average of 16 months. Eight-eight percent indicated they interact daily or hourly with their team members.

#### Measures

Participants were asked to describe the purpose of their primary team, their role assignment, and how frequently they interacted with team members. After this reflection, team accountability was measured with the TAQ (*α* = 0.88), trust was measured with four items (Simons & Peterson, 2000), and team commitment was measured with five items (Kirkman and Rosen, 1999). We selected trust and commitment for comparison constructs because they are the most closely related to team accountability and we wanted to ensure the TAQ accounted for variance beyond these related constructs. All items were measured using a 7-point scale where 1 = disagree strongly and 7 = agree strongly.

#### Analyses

A series of CFAs were conducted in MPlus. Consistent with previous scale development best practices (e.g., Chen et al., 2005; Hinkin, 1998) we first conducted a confirmatory factor analysis that placed all eight items of the TAQ on one factor. The CFA revealed a single-factor structure that provided an excellent fit for the data, χ^2^(15) = 20.77, n.s., comparative fit index (CFI) = 0.99, and standardized root mean square residual (SRMR) = 0.02 (Hu & Bentler, 1999). Per Table 3, all items of the TAQ exceed the 0.30 cut-off value (Hair et al., 2009) and significantly loaded onto a single factor. The TAQ items exhibit a mean inter-item correlation of 0.51. These results provide evidence of a single-factor structure for the TAQ, supporting the previous EFA results.

**Table 3:** Confirmatory factor analysis of TAQ (Study 1c)

		Factor Loading
1	We are accountable to each other as a team for our performance	.79
2	We are required to justify or explain our performance relative to team goals	.61
3	We hold each other accountable for doing our individual work	.80
4	We take responsibility as a group for our decisions	.74
5	In meetings we often explain to each other why we do certain things	.49
6	We monitor our team performance against our objectives	.63
7	We keep each other informed of our progress	.65
8	We can count on team members to deliver what they promise	.74

Factor loadings are standardized

##### Discriminant Validity

A two-factor model of team accountability and team trust (χ^2^_(53)_ = 143.83, CFI = 0.93, SRMR = 0.05) fits the data much better than a single-factor model (Δ χ^2^_(1)_ = 231.31, *p* < 0.001). Similarly a two-factor model of team accountability and team commitment (χ^2^_(64)_ = 163.06, CFI = 0.94, SRMR = 0.05) fits the data better than a single-factor model (Δ χ^2^_(1)_ = 176.198, *p* < 0.001).

##### Convergent Validity

As part of our CFAs, we also explored the correlation between latent variables and found the latent variables to be related but not overly so (i.e., TAQ & Trust, *r* = 0.59, *p* < 0.001; TAQ & Commitment, *r* = 0.77, *p* < 0.001). Taken together, these results suggest that team accountability as measured by the TAQ is a unique construct.

With a usable measure of team accountability in hand, we next tested our hypotheses regarding the emergence of accountability in teams and whether it predicts effort, performance, viability, and positive attitudinal states. To do so, we followed student teams competing in a business simulation for a span of three months.

### Study 2: Hypothesis Testing

Sixty-five teams of students at an Irish business school participated in this study. These teams were tasked with using a simulation platform to develop a virtual business and then to compete over several months with fellow teams for market share and profits (www.marketplace-simulation.com). Participants were randomly assigned to a team. Membership was further stratified by gender and nationality to ensure each team included a randomly assigned woman and international student. All teams had 4 to 5 members except one team with only 3 members. These teams operated under identical formal accountability structures, were given the same team task, started and worked together for the same amount of time, and had similar resources at their disposal. Naturally controlling for these contextual features was deemed more valuable in testing our hypotheses about collective accountability than the external validity gained by conducting our research in a professional setting, particularly when business students have the requisite understanding to respond knowledgeably to basic issues such as their own team dynamics (Bello et al., 2009). The average amount of time spent by the team on the stimulation platform was 68 hours (h)—summed across team members—with additional time spent on preparation, report writing, weekly tutorials, team meetings, and presentations.

Of the 302 individuals working on these teams, we received responses from 268 individuals over the course of the study. However, only 253 individual responses were usable after removing participants due to incomplete data, missing consent forms, and/or failed attention checks. Most participants provided data at one or two waves rather than all three, reflecting in-person attendance trends in the module.

#### Design

Each team was required to provide a written weekly report on its progress and member roles and individual contributions. Further formal accountability processes include monitoring of performance via the simulation platform, weekly discussion with a faculty expert, and a video recorded final presentation to two faculty experts. Of note is that the accountability expectations, timelines, and evaluation criteria were the same for each team in the competition. This context should ensure that any variability in accountability that remains is primarily due to internal team dynamics as opposed to formal structural accountability mechanisms.

Three waves of data were collected. The lead author distributed hardcopy study materials at the beginning of a weekly tutorial and returned after the end of each session to collect completed questionnaires. Time 1 occurred during the initial development of the team in February 2016. Time 2 occurred at the midpoint of the simulation in March 2016. At that point, teams had completed significant work together and had also received substantial feedback on their team performance. Time 3 occurred in April 2016 near the conclusion of the simulation. At that stage, there was little teams could do to change their performance on the simulation, but they were still collaborating on a presentation to a panel of experts. After the conclusion of the competition, team performance data were recorded from the simulation platform.

#### Measures

Previously used measures were used for data collection. Accountability was measured using the 8-item TAQ.^1^ Affective *identification* and *collective efficacy* were measured shortly after the start of the project. Intragroup *trust* and team *commitment* were measured at the start, midpoint, and end of the team performance cycle. The *viability* of each team was measured at the end of the project.

We used financial performance in the simulation to operationalize overall *team performance*. The simulation platform measured financial performance as net profit from current operations divided by the total shares issued. A positive and large number is optimal. The simulation ran over 8 financial quarters. At the end of the fourth quarter, halfway through the simulation, teams received their first financial scorecard for their new business enterprise. At this time, the average was -0.8 across all 65 teams (*min* = -22.0, *max* = 89.0, *SD* = 15.7). Average team performance in the final quarter of the stimulation was 62.5 (*min* = -14.9, *max* = 431.2, *SD* = 82.4). Due to skewness and kurtosis, the square root of the data is used in our analyses of financial performance. Several teams operated at a loss, so a constant was added to the original data to ensure the lowest value was equal to one prior to taking the square root.

*Effort* as a measure of team effectiveness was measured as the time the team invested in the task. The simulation platform collected this data through the time each team member spent actively on the platform. We divided time spent on the platform by team size to account for the number of persons available to contribute to the task. During the fourth quarter, the average team member was spending 12.0 h on the platform (*min* = 4.5 h, *max* = 22.5 h, *SD* = 4.0 h), and during the final quarter, the average team member was spending 10.6 h on the platform (*min* = 1.8, *max* = 26.8, *SD* = 5.7). A logarithmic transformation was implemented as the data were markedly positively skewed.

#### Analyses

SPSS was used to test our hypotheses. Analyses are reported at the level of the team unless noted otherwise. The rationale for this approach is that we are predicting team-level outcomes from team-level inputs as that matches our theoretical viewpoint of team accountability. We recognize that there is room for debate over whether this is the most accurate way to represent accountability at the beginning of the team lifecycle as some teams may have crystalized collective perceptions more quickly than others; however, our measurement and conceptualizations were at the team level so we report those analyses. Also, in the study environment teams received clear, uniform, and periodically reinforced formal accountability expectations which should have accelerated the sharedness of group accountability perceptions. In general, we found that relationships between constructs are stronger at the individual level than at the team level, so using team-level data yields more conservative estimates. Table 4 reports descriptive statistics and Table 5 provides details on reliability and variance statistics for all self-report measures.

**Table 4:** Descriptive statistics (Study 2)

	Mean	*SD*	*N*	1	2	3	4	5	6	7	8	9	10	11	12	13	14	15
*Time 1*																		
1. Accountability	5.83	.38	60															
2. Trust	5.96	.56	59	.67**														
3. Commitment	6.25	.43	59	.45**	.55**													
4. Identification	6.13	.59	59	.58**	.65**	.61**												
5. Collective Efficacy	6.17	.44	59	.55**	.53**	.65**	.71**											
*Time 2*																		
6. Accountability	5.82	.55	58	.25	.10	.25	.21	.22										
7. Performance	4.47	1.48	65	−.07	−.08	−.13	.03	−.02	.08									
8. Effort	2.17	.17	65	.26*	.02	−.13	.12	.10	.15	.19								
9. Trust	6.03	.64	58	.16	.07	.36**	.34*	.31*.	.74**	.00	.05							
10. Commitment	6.29	.64	58	.11	.02	.28*	.29*	.25	.67**	.12	.14	.85**						
*Time 3*																		
11. Accountability	5.88	.63	55	.06	−.04	.18	.16	.13	.29*	−.01	.21	.34*	.35*					
12. Performance	7.96	3.90	65	.09	−.20	−.23	−.11	−.13	.25	.44**	.41**	.10	.24	.24				
13. Effort	2.08	.25	65	.25	−.07	−.03	.05	−.03	.32*	.13	.40**	.05	.08	.19	.42**			
14. Viability	5.78	.82	60	.18	.02	.11	.22	.11	.45**	.13	.35**	.35**	.33*	.67**	.32*	.29*		
15. Trust	5.82	.77	60	.22	.17	.15	.31*	.15	.39**	−.11	.18	.40**	.38**	.73**	.22	.11	.78**	
16. Commitment	6.12	.75	60	.04	−.03	.11	.10	−.06	.47**	.01	.14	.40**	.40**	.62**	.27*	.17	.81**	.72**

Performance is measured as the square root of net team profit from final quarter operations divided by the number of stock shares issued. Effort is measured by the total minutes spent by the team on the simulation during the final quarter, adjusted for the size of the team and subject to a logarithmic transformation

*Correlation is significant at the 0.05 level (2-tailed)

**Correlation is significant at the 0.01 level (2-tailed)

**Table 5:** Variance and reliability statistics (Study 2)

		grandmean	variance between	variance within teams							
Individual reports at:	*α*	(*γ*)	teams (*σ*^2^_B_)	(σ^2^_W_)	*ICC*	Wald *Z*	*p* <	avg *r*_wg(*j*)_ uniform	> .70	avg *r*_wg(*j*)_ slight skew	> .70
*Time 1*											
Team Accountability	0.72	5.70	0.03	0.44	0.06	0.95	0.35	0.92	98%	0.95	98%
Team Trust	0.81	6.00	0.01	0.63	0.01	0.17	0.87	0.90	94%	0.85	88%
Team Commitment	0.84	6.28	0.03	0.37	0.06	1.01	0.31	0.95	98%	0.93	96%
Team Identification	0.85	6.19	0.09	0.50	0.13	1.82	0.07	0.93	100%	0.90	90%
Collective Efficacy	0.81	6.22	0.04	0.31	0.10	1.50	0.13	0.97	98%	0.94	98%
*Time 2*											
Team Accountability	0.80	5.64	0.16	0.37	0.23	2.58	0.01	0.91	93%	0.86	91%
Team Trust	0.87	6.01	0.15	0.71	0.15	1.83	0.07	0.89	91%	0.83	87%
Team Commitment	0.88	6.28	0.21	0.50	0.23	2.69	0.01	0.92	93%	0.87	89%
*Time 3*											
Team Accountability	0.78	5.86	0.09	0.41	0.15	1.42	0.16	0.93	95%	0.89	93%
Team Viability	0.92	5.84	0.34	0.57	0.27	2.89	0.00	0.93	96%	0.88	89%
Team Trust	0.87	5.90	0.21	0.69	0.19	1.92	0.06	0.87	85%	0.78	80%
Team Commitment	0.90	6.17	0.18	0.59	0.19	2.03	0.04	0.90	91%	0.84	87%

Note: Grand mean is the average level of a construct within the teams. The intra-class correlation coefficient (ICC) reports the proportion of variance in scale that lies between teams. Wald Z tests if there is signficant variation across teams. *r*_wg(*j*)_ is an index of agreement within team members for *j* items on the multi-item measure. Out of range values were reset to zero. A uniform null distribution for item responses is a rectangle (i.e., 1 and 7 equally likely to be chosen), but a slightly skewed distribution is more appropriate for scales such as these with a leniency bias

Because we believe collective accountability to be an emergent state, we first explored the stability of collective accountability across time (Marks et al., 2001; Waller et al., 2016). To do this we retained the 46 teams for which we had data at all three collection points. Although there are significant differences in overall accountability perceptions between teams (*β* = *5.83, SE* = 0.08*, t* = 74.95, *p* < 0.001), the descriptive statistics indicates that average accountability across the subsample of 46 teams with complete data does not vary much with time, i.e., *Time 1 Mean* = 5.83, *Time 2 Mean* = 5.83 and *Time 3 Mean* = 5.88. An evaluation of the accountability within these 46 teams indicates that neither the linear nor quadratic polynomial growth rates are significant in explaining team changes in accountability over time. Random coefficient modeling with time as a repeated measurement at Level 1 and team membership as a Level 2 effect suggests that accountability does not appear to change much over time within teams. Bliese (1998, 2000) argues that the ICC(2) is useful for detecting emergent states. The ICC(2)s were 0.67 at Time 1, 0.82 at Time 2 and 0.85 at Time 3, exhibiting increasing consistency in evaluations over time. Taken together, these exploratory analyses suggest that *within* a team accountability perceptions are relatively stable.

#### Hypotheses

Hypothesis 1 states that initial team accountability during team development should emerge hand in hand with team (a) trust, (b) affective identification, (c) commitment, and (d) collective efficacy. The data indicate a strong positive relationship between initial team accountability perceptions and trust (*r* = 0.67), commitment (*r* = 0.45), affective identification (*r* = 0.58), and collective efficacy (*r* = 0.55) during the initial stages of team collaboration. The first hypothesis is therefore supported. Formative team accountability perceptions are part and parcel of other positive emerging team attitudes.

Our next set of hypotheses examined the ability of ‘established’ accountability at the project midpoint to predict subsequent team outcomes. We assumed that end point values of the dependent variables are in part a function of their midpoint values at time 2. For example, final performance is in some ways constrained by midpoint performance. Although including lagged dependent variables often decreases coefficient estimates for the independent variable of primary interest and has been argued to unduly suppress the explanatory power of the independent variable, we included interim trust, commitment, performance, and effort as lagged dependent variables to provide a less biased test of the hypothesized relationship between accountability and subsequent team effectiveness and attitudes (Keele & Kelly, 2006). Lagged trust and commitment are highly correlated with another (*r* = 0.85), therefore we added them together in a single linear composite to reduce collinearity. Regression results are reported in Table 6.

**Table 6:** Regression analyses (Study 2)

	*Time 3*				
	Performance	Effort	Viability	Trust	Commitment
	(H2)	(H3)	(H4)	(H5a)	(H5b)
Model 1					
*Dependent variables (Time 2)*					
Performance	.37***	.05	.02	-.17	-.05
Effort	.44***	.41**	.25†	.18	.06
Trust + Commitment	.11	.03	.33*	.39**	.41**
*R*^2^	.40***	.18*	.19*	.22**	.18*
Model 2					
*Dependent variables (Time 2)*					
Performance	.36***	.04	.00	-.18	-.08
Effort	.43***	.37**	.24†	.17	.06
Trust + Commitment	-.01	-.34†	.06	.25	.15
*Independent variable (Time 2)*					
Accountability	.17	.51**	.38*	.21	.36*
*R *^2^	.41***	.29**	.26**	.24**	.24**
Δ*R*^2^	.01	.12**	.07*	.02	.06*

The above regression coefficients are all standardized

***p < .001, ** *p* < .01, * *p* < .05, ^†^*p* < .10

*n* = 58 teams (H2 and H3); *n* = 53 teams; (H4, H5a, and H5b)

Hypotheses 2 and 3 predict positive effects of team accountability on team performance and effort. Hypothesis 2 does not receive strong support. Our data find that accountability is positively related to subsequent team performance, but is not a statistically significant predictor when added to a model that includes midpoint performance (*β* = 0.17, *R*^2^ = 0.41, Δ*R*^2^ = 0.01, n.s.). The strongest predictors of team performance are previous performance (*β* = 0.36) and effort (*β* = 0.43). In support of Hypothesis 3, we found team accountability is associated with a significant increase in team effort (*β* = 0.51, *R*^2^ = 0.29, Δ*R*^2^ = 0.12, *p* < 0.01).

The data support Hypothesis 4, that team accountability is associated with greater viability (*β* = 0.38, *R*^2^ = 0.26, Δ*R*^2^ = 0.07, *p* < 0.05). Support for Hypothesis 5 is mixed. Accountability is positively associated with later trust (H5a) but this effect is not statistically significant (*β* = 0.21, *R*^2^ = 0.24, Δ*R*^2^ = 0.02, n.s.) when added to model with midpoint trust and commitment. The strongest predictor of trust is previous trust and commitment (*β* = 0.39). Although support is limited for Hypothesis 5a, Hypothesis 5b is supported: midpoint team accountability predicts subsequent team commitment (*β* = 0.36, *R*^2^ = 0.24, Δ*R*^2^ = 0.06, *p* < 0.05).^2^

## Discussion

Our approach follows a relational understanding of accountability because we position team accountability as an emergent understanding of how things are done on a team that depends in large part on multiple evolving subjectivities rather than formal accountability structures. We agree with the view that a team is a ‘forum’ for moral tensions that often must be worked through by the members themselves (Sewell, 2012). Our data suggest that differences can emerge between teams even when they operate in a highly structured accountability environment, report to the same authority figures, share the same geographical location, work on identical tasks, and are comprised of members of similar experience.

Following a team over time allows us to develop a construct of team accountability that embraces the social processes through which ‘thick’ ethical norms are socially constituted (Islam & Greenwood, 2021). During the early life of a group, team accountability is closely related to trust, commitment to getting the job done, identifying as a team, and the belief that the team can accomplish what it sets out to do. This constellation of positive attitudes persisted as the team evolved. It appears the lived experience of teamwork is of better quality when felt accountability is higher. Perhaps the strongest proof in the value of team accountability lies in members being keener to continue to collaborate after the end of the project.

Two results were unexpected. First, collective team accountability did not predict trust when we included lagged variables. Our inquiry was not intended to identify the strongest predictor of trust, but rather to test whether accountability measurably increased subsequent trust. One explanation for the lack of a relationships is statistical, namely that interim trust and accountability are highly correlated predictors and this makes it difficult for accountability to add predictive value in the hierarchical model. A more theoretical explanation is that once teams are up and running, trust is established and the strongest predictor of future trust is trust itself.

A second unanticipated result is that accountability did not meaningfully predict financial performance when previous performance and effort were included as predictors. Fostering a sense of collective accountability is often intended as a managerial tool for driving organizational objectives and reducing loafing behaviors. In our study, team accountability exhibited stronger ties to emergent and affective team states than to subsequent performance. In other words, accountability expectations are not a performance panacea, although they do encourage greater time investment in shared goals and precede measurable improvements in some dimensions of concrete task performance.

It is possible our results were limited by the number of teams in our sample size or by using a single number to encapsulate performance, but our interpretation is that ultimate performance is often somewhat *outside* the control of the team (de Leede et al., 1999). In the simulation for example, financial performance depended in part on decisions of competitors and market conditions. Work analyzing the ethics of teamwork have touched on this concern previously, for example highlighting violations of distributive justice where rewards are not in-line with collective efforts (Sewell, 2012).

### Theoretical Contribution

Accountability is a sprawling theme that crosses disciplinary and methodological boundaries. The primary contribution of this study is to weave together philosophical ethics, management, and social psychology theories to explain the emergence, coherence, and consequences of teams developing a sense of accountability. By uniting a philosophical understanding of relational accountability (Painter-Morland, 2006, 2007) with work on felt accountability (Hall et al., 2017) and emergent team states (Waller et al., 2016) we offer a perspective on accountability that is firmly situated in team dynamics. Our work grounds high level concepts of relational accountability in specific instances of team identification, efficacy, commitment, viability, and trust. Using contemporary philosophical thought to analyze team accountability introduces powerful ideas about the moral obligations and being accountable ‘toward’ rather than ‘for’.

Detailed learning about team accountability matters because autonomous and empowered teams are increasingly seen as the basic building block of agile organizations (Deloitte, 2020) and yet the existing accountability literature predominately views accountability as top-down compliance and formal mechanisms of control. A search of the *Journal of Business Ethics* in 2020 surfaced 183 abstracts for original research on accountability published since 1982. Over half of this work has been published in the past 10 years, suggesting an increase in scholarly interest. However, only two of these articles specifically examined ethical issues related to the accountability of teams (de Leede et al., 1999; Joosten et al., 2014).

Two practical limitations to understanding the intricacies of team accountability are the lack of an appropriate measurement instrument and the use of phantom or ephemeral teams (Hall et al., 2017; Kou & Stewart, 2018). Our work supports future cross-disciplinary research on collective accountability by piloting and validating the TAQ. We demonstrate that this measure is reliable across three distinct work environments (the USA, Republic of Ireland, the United Kingdom), and use our findings to anchor accountability to the fundamental dynamics of a team. Another contribution to the study of accountability is to examine accountability in its natural environment, such as we did with the pharmaceutical leaders in Study 1b and the longitudinal study in Study 2. Most prior team accountability research has been conducted in experimental settings and few studies have utilized teams engaged in a task for real reward and punishment, and none to our knowledge have explored accountability over the life of the team.

### Practical Implications

Our findings highlight that even within a highly structured accountability environment, accountability varies. Accountability policies and procedures are filtered through a subjective lens so managers should not expect teams to react identically to the same policies and procedures. For example, formal accountability features are often open to misinterpretation despite frequent verbal and written cues (Patchan et al., 2018). Relationships among teammates are therefore critical to understanding team accountability.

Our findings also suggest that initial levels of group accountability persist, so managers should support initial team interactions because they may count ‘extra’ in forming shared perceptions. If you seek a direct lever to improve team performance, look elsewhere, particularly when future performance is contingent on performance at previous levels. Accountability appears to serve the team best by increasing effort, viability, and deepening commitment.

### Limitations and Future Directions

This study maps new territory and as such merits refinement and extension. We examine the relationship between relational accountability and team dynamics which is an essential step, but developing team accountability as a ‘thick’ rather than ‘thin’ ethical concept requires being descriptive *and* evaluative (Islam & Greenwood, 2021). Going forward, qualitative enquiries would be well suited to examining what might foster or derail team accountability. Future prescriptive work could carefully examine the general assumption that perceived accountability is morally ‘good’ and that a team without shared accountability is ‘bad’. Our study simplistically presumes more accountability is superior, but theoretical work is needed to identify when robust team accountability perceptions are procedurally unfair or can turn toxic to the welfare of the team, for example through stress responses, interpersonal conflict, and shunning or other punishment of norm violators. Such lines of inquiry could also examine when divergent perceptions of accountability are better for team outcomes than convergent perceptions, for example when a team is making sense of a failure to perform or processing decisions with ethical implications.

We also call for expanded outcome variables beyond the usual suspects. Based on research that connects workplace cultures of love and anger to employee loneliness (Ozcelik & Barsade, 2018), we believe future research should explore whether team accountability might have mental health benefits including increased feelings of belonging and reduced loneliness at work, particularly in the age of virtual collaboration. The connection between relational accountability and these well-being related outcomes is a reminder that examining what is ‘good’ for members’ mental health is an important ethical dimension missing from most team outcome variables. Techniques such as team interviews, observations, diary studies, and analyses of team artifacts would be particularly well suited to examining pivotal events and relationships that might foster or derail team accountability.

Future theory building could build bridges between shared team accountability and the formalistic or utilitarian ethical orientation of a team (Adler et al., 2021; Pearsall & Ellis, 2011), team ethical culture and climate (Cabana & Kaptein, 2019; Kim & Vandenberghe, 2020), and collective virtues such behavioral integrity (Palanski et al., 2011). Whether team accountability is a virtue or vice for a team likely depends upon what sort of team it is, and whether possessing this disposition makes it better or worse as that kind of group (Byerly & Byerly, 2016). Our work joins these in responding to the plea to situate the ethics of teamwork in the activities and multiple subjectivities of the team, rather than in abstract moral concepts (Sewell, 2007).

Unraveling the connection between leadership and collective accountability is also worth exploring. We utilized self-managed teams in which leadership emerges organically. However, in organizations with hierarchical structures and leader/subordinate relationships, team accountability may arise as a substitute for strong team leadership, or strong leadership may be the driving force behind initial differences in team accountability perceptions. Furthermore, teams are likely to develop different types of accountability perceptions depending on the quality of their relationship with an external leader. For example, when mutual respect is absent or lost, a leader is more likely to lean on formal control mechanisms. In response, the team may limit accountability perceptions to the obligations specified. In contrast, when a team and leader form an emotional attachment and have positive mutual regard, accountability perceptions are more likely to be internalized (Erdogan et al., 2004). Regardless of formal accountability measures, in this situation perceived accountability should expand to encompass collaborative activities that contribute to mutual goals and well-being. A relational understanding of team accountability should therefore explore leader-member exchanges and their formative role in perceptions of how work should be carried out by the team.

Accountability as a shared team characteristic has been explicitly incorporated into our theorizing and the subsequent measurement strategy is aligned with this conceptualization, i.e., the TAQ is based on a referent shift model and many of our analyses use the team mean (Chan, 1998). This approach is consistent with how the majority of emergent team states are conceptualized and operationalized (Waller et al., 2016), but future work could study relational team accountability through alternate models of emergence (Chan, 1998; Kozlowski & Klein, 2000). For example, analyses of variation instead of convergence in team accountability perceptions could indicate active fault lines, healthy disagreement about the necessity of accountability processes, or insufficient team experiences to form the basis of shared perceptions. To further understand the relational nature of team accountability, researchers could examine developmental patterns of accountability perceptions across a team and the extent to which they reflect networks of trust, friendships, and moral obligations.

A strength of our research design is that we are able to examine accountability over the team’s lifespan in a highly controlled situation with fixed external accountability measures and leadership, identical performance challenges and timelines, objective measurement of performance and effort, and by teams of similar demography, size and experience. The effects of these factors would have been difficult to control for in a corporate setting, but the simulation and academic context we use cannot realistically mirror the intensity of ‘real world’ accountabilities. Team accountability may well be reliably predictive of task performance when performance accompanies meaningful financial and professional implications or is measured along multiple dimensions rather than reduced to a single metric as in this study. Future research should explore the connection between team accountability and performance in contexts with higher stakes.

## Conclusion

This paper unites concepts from relational and felt accountability and team dynamics to provide an initial explanatory framework of team accountability and a measurement instrument that can be used in practice and research to continue this work. If accountability is the glue that holds together organizations, and teams are the organizational unit of necessity and choice, then perceptions of team accountability are worthy of intense attention across disciplines.

## Appendix

See Table

**Table 7:** Origins of team accountability items (Study 1a)

Published Accountability Measure	Individual Item	Piloted Team Accountablity Item (**retained items are in bold lettering**)
*greater accountability* first factor of multi-item accountability instrument based on interviews of managers' experiences with accountability; 4-item scale (Ogden et al., 2006, p. 537)	1. I feel more exposed as a manager now that assessments of how well I perform against targets are easier to make.	Assessments of how well we perform as a team makes us feel open to criticism.
	2. I am now more accountable for my performance. *(second highest loading item)*	**We are accountable to each other as a team for our performance**.
	3. I check up much more now on how well I am doing against the business targets I have to achieve.	We frequently check how well we are doing against our team targets.
	4. The amount of monitoring of performance against targets has increased. *(highest loading item)*	**We monitor our team performance against our objectives.**
*perceived accountability for task performance* extent to which employees perceive themselves as accountable for achieving a prescribed level of task performance; 3-item scale (Mero et al., 2014, p. 1633, 1639)	1. Others in my organization can observe the outcome of my work performance in terms of achieving unit goals.	Others in my team can observe the outcome of my performance relative to team goals.
	2. In my organization achieving unit goals is directly attributed to an individual’s personal actions.	Our joint actions contribute to achieving team goals.
	3. I am required to justify or explain my performance in terms of achieving unit goals.	**We are required to justify or explain our performance relative to team goals.**
*felt accountability* an implicit or explicit expectation that one's decisions or actions will be deemed important or noteworthy, and will be subject to evaluation by salient others with the belief that there exists the potential for one to receive either rewards or sanctions; 8-item scale (Hochwater et al., 2005, p. 26)	1. Co-workers, subordinates, and bosses closely scrutinize my efforts at work.	[Classmates and students] who are not on the team closely scrutinize our team efforts on this project.
	2. I often have to explain why I do certain things at work.	**In meetings we often explain to each other why we do certain things.**
	3. In the grand scheme of things, my efforts at work are very important.	In the grand scheme of things, our efforts as a team are very important to us.
	4. If things at work do not go the way they should, I will hear about it from top management.	Our team would talk to a team member if his or her work is disappointing.
	5. The jobs of many people at work depend on my success or failures.	The [grades] of team members depend on our success as a team.
	6. To a great extent, the success of my immediate work group rests on my shoulders.	To a great extent, the success of our team is in our hands.
	7. I am held very accountable for my actions at work.	**We hold each other accountable for doing our individual work.**
	8. Top management holds me accountable for all of my decisions.	**We take responsibility as a group for all our decisions.**
*team accountability* team members’ expectations of being held answerable for their common actions or decisions, arises from ongoing interactions in which formal accountability mechanisms are interpreted and internalized	(items were written for this study)	On our team it is easy to get away without doing a fair share of the work. People who make an extra effort on behalf of the team are recognized.
		**We can count on members to deliver what they promise.**
		We expect to let each other know when we cannot keep a commitment.
		**We keep each other informed of our progress.**
		When someone on the team lets us down we confront them.
*procedural accountability* manipulation checks for decision process accountability condition in lab experiments (Pitesa & Thau, 2013, p. 553, 534)	1. When you made your decision, did you believe you would have to justify the process of reaching the decision to researchers?	Our team expects to have to explain our decision processes to [supervisor].
	2. Will your decision be assessed focusing on the decisionmaking procedures?	Our team focuses on the joint decision making procedures we use.
*process accountability* results when buyers expect others to evaluate the quality of their decisionmaking process (Doney & Armstrong, 1996, p. 59)	1. I felt accountable to others for the quality of the process I used regarding this decision.	We are accountable as a team for the quality of the decision processes we use.
	2. I thought that others would evaluate the procedures I followed with respect to this purchase.	Within our team we evaluate the procedures we follow in making decisions.
*outcome accountability* manipulation checks for manipulation of accountability for decision results in lab experiments (Pitesa & Thau, 2013, p. 553, 534)	1. When you made your decision, did you believe you would have to justify the outcome of your decision to researchers?	Our team expects to have to explain the outcomes of our decisions to [supervisor].
	2. Will your decision be assessed focusing on the outcome of the decision?	Our team focuses on [the supervisor's] evaluations of our work.

7.

## Footnotes

1 Following Chen and colleagues’ framework for validating team-level constructs (2003) we conducted a team-level CFA on the TAQ using Time 3 data. The results indicate adequate support for the unitary factor structure of the TAQ at the team level, χ2(19) = 31.05, *p* = .04; CFI = 93; SRMR = .08.

2 Supplementary analyses without lagged dependent variables yields notably stronger effects. In particular, the regression coefficient for team accountability predicting final team performance (H2) increases from *β* = .17 to *β* = .25 and approaches traditional statistical significance at *p* < .06. The regression coefficient for team accountability predicting team trust (H5a) increases from *β* = .21 to *β* = .39 and achieves statistical significance at *p* < .01.
